# The Second Mitochondria‐Derived Activator of Caspases Mimetic BI 891065 in Patients With Advanced Solid Tumors: Results From Two Phase I Studies

**DOI:** 10.1002/cam4.71451

**Published:** 2025-12-17

**Authors:** Manish R. Patel, Erika P. Hamilton, Ben George, Gunther Kretschmar, Akiko Harada, Ralph Graeser, Anastasia Eleftheraki, Yoshifumi Tachibana, Noboru Yamamoto

**Affiliations:** ^1^ Florida Cancer Specialists/Sarah Cannon Research Institute Sarasota Florida USA; ^2^ Sarah Cannon Research Institute Nashville Tennesse USA; ^3^ Medical College of Wisconsin Milwaukee Wisconsin USA; ^4^ Boehringer Ingelheim Pharma GmbH & Co. KG Biberach an der Riss Germany; ^5^ Nippon Boehringer Ingelheim Co., Ltd. Kobe Japan; ^6^ Astra Zeneca Barcelona Spain; ^7^ Nippon Boehringer Ingelheim Co., Ltd. Tokyo Japan; ^8^ National Cancer Center Hospital Tokyo Japan

**Keywords:** checkpoint control, clinical cancer research, experimental therapeutics, ezabenlimab, IAP, medical oncology, PD‐1, SMAC mimetic, solid tumors, target therapy

## Abstract

**Introduction:**

BI 891065, a second mitochondria‐derived activator of caspases mimetic targets the inhibitor of apoptosis (IAP) family member cIAP1. We describe two first‐in‐human phase 1 trials assessing BI 891065 ± the anti‐programmed cell death protein‐1 (PD1) antibody, ezabenlimab, in advanced solid tumors.

**Methods:**

Trials were conducted in the USA (NCT03166631) and Japan (NCT04138823). Dose escalation of BI 891065 monotherapy (part A) and combined with ezabenlimab (part B) was guided by a Bayesian Logistic Regression Model with overdose control. Primary endpoints were maximum tolerated dose (MTD) and number of patients with dose‐limiting toxicities (DLTs) in Cycle 1. Other endpoints included objective response (RECIST v 1.1), pharmacokinetics, and changes in peripheral blood mononuclear cell (PBMC) and tumor cIAP1 levels.

**Results:**

Twenty‐five patients (USA study) received 5–400 mg daily BI 891065 monotherapy; 12 patients (Japan study) received 100 mg daily, 200 mg daily, or 200 mg twice‐daily BI 891065 monotherapy. No DLTs occurred in the USA study; three occurred in the Japan study: grade 3 increased bilirubin (*n* = 2) and maculopapular rash (*n* = 1). Neither study reached MTD for monotherapy. Treatment‐related adverse events (TRAEs) occurred in 52% and 75% of patients, respectively. BI 891065 plus ezabenlimab combination (USA study part B only) was received by 37 patients (50–400 mg daily, 200 mg twice‐daily) plus ezabenlimab (240 mg fixed‐dose, Day 1 of 21‐day cycles). One DLT occurred (grade 2 pneumonitis). MTD was not reached. TRAEs occurred in 81% of patients. Neither study reported objective responses; 25%–40% and 35% of patients achieved stable disease with BI 891065 monotherapy and the combination, respectively. cIAP1 levels were reduced in PBMCs and biopsies.

**Conclusions:**

BI 891065 was tolerable in patients with advanced solid tumors, demonstrating target engagement as monotherapy and combined with ezabenlimab. Both studies ended early due to efficacy data that were insufficiently promising to justify continuation.

**Trial Registration:**

NCT03166631, NCT04138823

## 1 Introduction

Although immune checkpoint inhibition has demonstrated promising results across a range of human malignancies, up to 80% of patients with previously treated advanced solid tumors do not respond to checkpoint inhibitor monotherapy [1, 2, 3]. As a result, effective treatment for advanced cancers is an unmet need for many patients.

The inhibitor of apoptosis (IAP) family comprises regulatory proteins involved in immune signaling [4]. Three of the IAP family members—cIAP1, cIAP2, and XIAP—promote cell survival in cancer cells in response to cytotoxic agents independent of immune cell signaling. This is associated with poor prognosis [5, 6]. IAPs have some endogenous antagonists, including the second mitochondria‐derived activator of caspases (SMAC) protein, which binds to and promotes the degradation of IAPs, particularly cIAP1 [5]. In preclinical studies, small‐molecule SMAC mimetics have been shown to induce cytokine‐mediated cell death upon binding to IAPs in susceptible tumors [7]. Moreover, targeting of cIAP1 appears to reduce metastatic spread by inhibiting the ability of tumor cells to cross the vascular endothelium during extravasation [8].

Several SMAC mimetics such as birinapant, LCL161, and DEBIO1143, which all inhibit cIAP1, cIAP2, and XIAP with varying potencies, have been assessed in early clinical trials [5, 9, 10, 11, 12]. These studies were largely disappointing, with limited activity as monotherapies against solid and hematologic tumors. Furthermore, some of these agents were associated with dose‐limiting cases of cytokine‐release syndrome. This probably reflects the observation that inhibition of IAPs, particularly XIAP and XIAP deficiency in general, is associated with increased production of pro‐inflammatory cytokines, including tumor necrosis factor alpha (TNFα) [5, 9, 10, 11, 13].

BI 891065 is a novel, potent SMAC mimetic that has demonstrated selectivity for cIAP1 (half‐maximal inhibitory concentration [IC50]: ≤ 1 nM) over cIAP2 (IC50: 15 nM) and XIAP (IC50: 206 nM) in cell‐based assays [14]. In syngeneic mouse tumor models, BI 891065 potently induced immunogenic cell death, which was further potentiated when combined with immune checkpoint inhibitors. Thus, there is rationale for assessing BI 891065 with and without anti‐PD‐1 antibodies in clinical studies [14, 15]. Ezabenlimab (BI 754091) is a humanized programmed cell death 1 (PD‐1)‐targeting monoclonal antibody [16] that has demonstrated preliminary antitumor activity and a manageable tolerability profile in patients with solid tumors, both as monotherapy and in combination with other agents [17, 18, 19].

This paper describes the results of two independent phase 1 dose‐finding studies that evaluated BI 891065 as monotherapy and in combination with ezabenlimab, in patients with advanced solid tumors.

## 2 Methods

### 2.1 Design of Studies

These were phase 1, open‐label, dose‐finding studies conducted in the USA (NCT03166631) and Japan (NCT04138823). Both studies had multiple parts planned: part A, dose escalation of BI 891065 monotherapy in advanced/refractory solid tumors; and part B, dose escalation of BI 891065 plus ezabenlimab combination therapy in patients with specific advanced/refractory solid tumors (bladder, colon, breast, non‐small cell lung cancer [NSCLC], ovarian, pancreatic, renal, esophagogastric, sarcoma, prostate, and melanoma). NCT03166631 had a planned third part (part C), an expansion cohort of BI 891065 plus ezabenlimab combination therapy in patients with NSCLC who had failed PD‐1/PD ligand 1 treatment, but the study was discontinued before part C commenced. NCT04138823 was discontinued before part B.

The studies were conducted in accordance with the Declaration of Helsinki, the International Council for Harmonization of Technical Requirements for Pharmaceuticals for Human Use Good Clinical Practice guidelines, and local regulations. Both studies were approved by the local ethics committees of participating centers. All patients provided written informed consent before participation in any trial procedures.

### 2.2 Patient Population

Enrolled patients had a confirmed diagnosis of advanced, unresectable and/or metastatic solid tumors that had failed on or were ineligible for standard treatment or for which no therapy of proven efficacy existed. Other key inclusion criteria were: age ≥ 18 years or being of legal age, according to local legislation at screening; Eastern Cooperative Oncology Group performance status of 0 or 1; life expectancy of ≥ 12 weeks after the start of treatment based on investigator's judgment; presence of measurable lesion(s) according to Response Evaluation Criteria in Solid Tumors version 1.1 (RECIST v1.1); at least one tumor lesion amenable to biopsy; and willingness to undergo a biopsy prior to first treatment and another biopsy while on therapy unless clinically contraindicated (part B only). Part B included patients with specific advanced/refractory solid tumors, as noted in the previous section. The main exclusion criteria were: treatment with any other anticancer drug within 4 weeks or 5 half‐lives (whichever was first) prior to first administration of BI 891065; and serious concomitant disease or medical condition that could affect compliance with trial requirements, or which was considered relevant for the evaluation of the efficacy or safety of the trial drugs. Inclusion and exclusion criteria are listed in the Table S1.

### 2.3 Treatment, Endpoints and Assessments

In part A of the USA study (NCT03166631), patients received BI 891065 at dose levels of 5, 15, 25, 50, 100, 200, or 400 mg once daily (QD). Dose levels for part A of the Japan study (NCT04138823) were 100 or 200 mg QD, or 200 mg twice daily (BID). In part B of the USA study, patients received BI 891065 50, 200, or 400 mg QD or 200 mg BID, in combination with an intravenous infusion of ezabenlimab 240 mg on Day 1 of 21‐day cycles.

For both studies, the primary endpoints for part A were: (i) maximum tolerated dose (MTD) of BI 891065 defined as the highest dose with < 25% risk of the true dose‐limiting toxicity (DLT) rate being ≥ 33% during the end of the first treatment cycle (MTD evaluation period); and (ii) the number of patients with DLTs in the MTD evaluation period (Cycle 1). The definition of DLTs is provided in the Supporting Information.

Secondary endpoints for part A were: (i) the number of patients with DLTs observed during the entire treatment period; (ii) pharmacokinetic (PK) parameters of BI 891065 after single and multiple dosing, including maximum plasma concentration (C_max_) at steady state (C_max,ss_), area under the concentration–time curve (AUC) of the dosing interval at steady state (AUC_tau,ss_), and AUC from time 0 to the last quantifiable concentration (AUC_0–tz_); and (iii) objective response (OR) based on RECIST v1.1 as assessed by the investigator and/or the local radiologist. Other endpoints for part A included OR based on modified RECIST for use in trials testing immunotherapeutics (iRECIST) and the pharmacodynamic investigation of cIAP1 protein expression levels in peripheral blood mononuclear cells (PBMCs), assessed as percent change from baseline via an enzyme‐linked immunosorbent assay (ELISA).

For part B of the USA study, the primary endpoints were: (i) MTD of BI 891065 in combination with ezabenlimab; and (ii) the number of patients with DLTs in the MTD evaluation period (Cycle 1). Secondary endpoints were: (i) the number of patients with DLTs observed during the entire treatment period; (ii) PK parameters for both BI 891065 and ezabenlimab; and (iii) OR based on RECIST v1.1. Other endpoints included OR based on iRECIST and change from baseline of cIAP1 protein expression levels in tumor tissue via immunohistochemistry. Food effect on the PK of BI 891065 was an exploratory endpoint for patients who received BI 891065 QD. On Cycle 1 Day 15, patients received BI 891065 in a fasted state, and on Cycle 1 Day 16, patients received BI 891065 in a fed state following a standard continental breakfast (~688 kcal or 2880 kJ).

For both studies, assessments included: (i) tumor response (assessed at baseline, every 6 weeks for the first 6 months, and every 9 months thereafter until death), progressive disease, start of subsequent therapy, or end of trial; (ii) frequency, severity, and causal relationship of adverse events (AEs), tabulated by system organ class and preferred term, and coded using the Medical Dictionary for Drug Regulatory Activities and Common Terminology Criteria for Adverse Events version 5.0; (iii) hematology, biochemistry, coagulation, urinalysis, virology, and electrocardiogram tests; (iv) PK analysis of blood and urine samples at defined time points using validated assays; and (v) biomarker assessments (including alpha‐1 acid glycoprotein [AGP], and cytokines) at defined time points using validated assays. For the measurement of cIAP1 levels, PBMCs were collected at baseline, on Cycle 1 Day 1, 2, 8, and 15 during the treatment period, and at the end of treatment. Tumor biopsies for the measurement of cIAP1 degradation levels were taken at screening and after Cycle 1. Blood samples for cytokine assessment were taken on Days 1, 2, 8, and 15 (Cycle 1), and at the end of treatment. Details of the bioanalytical methods used are described in the Supporting Information.

### 2.4 Statistical Analysis

Dose escalation was guided by a Bayesian Logistic Regression Model (BLRM) with overdose control and MTD was considered reached if predefined criteria were fulfilled (see Supporting Information for further details). Based on BLRM simulation analyses, it was estimated that at least 20 (part A) and 30 (part B) evaluable patients should be treated in the USA study to determine the MTD with good precision. In the Japan study the estimate was at least 12 evaluable patients.

For the determination of the MTD, only MTD‐evaluable patients were considered. For the analysis of secondary and further endpoints, all patients treated with at least one dose of trial medication were included. All endpoints were summarized descriptively.

All analyses including PK were carried out using Phoenix WinNonlin version 8.1 and SAS software, version 9.4 (or later).

## 3 Results

### 3.1 Patients

In part A of the USA study, 25 patients received BI891065 QD (5 mg, *n* = 3; 15 mg, *n* = 1; 25 mg, *n* = 3; 50 mg, *n* = 4; 100 mg, *n* = 3; 200 mg, *n* = 3; and 400 mg, *n* = 8). Of these patients, 64% were female and the majority were Caucasian, with a median age of 69 years (Table 1). Nineteen patients (76%) had metastatic disease at diagnosis, and all patients had received prior systemic therapy; the median number of prior systemic therapies was 5 (range 1–9). Eleven patients (44%) had received prior immunotherapy.

**TABLE 1:** Patient demographics in the treated sets of the USA study (NCT03166631) and the Japan study (NCT04138823).

Characteristic	Study NCT03166631 (USA) Part A (*n* = 25)	Study NCT03166631 (USA) Part B (*n* = 37)	Study NCT04138823 (Japan) Part A (*n* = 12)
Gender, *n* (%)			
Male	9 (36)	16 (43)	4 (33)
Female	16 (64)	21 (57)	8 (67)
Median age, years (range)	69.0 (39–79)	64.0 (40–82)	52.5 (38–70)
Median height, m (range)	1.66 (1.50–1.91)	1.65 (1.47–1.83)	1.60 (1.41–1.75)
Median weight, kg (range)	77.1 (50.7–109.8)	75.2 (44.8–137.9)	52.9 (38.1–78.2)
Race, *n* (%)			
Black/African American	3 (12)	—	—
Caucasian	22 (88)	36 (97)	—
Asian	—	—	12 (100)
Other	—	1 (3)	—
ECOG PS, *n* (%)			
0	7 (28)	10 (27)	11 (92)
1	18 (72)	27 (73)	1 (8)
Metastatic disease at diagnosis, *n* (%)	19 (76)	24 (65)	11 (92)^a^
Prior surgery, *n* (%)	19 (76)	23 (62)	9 (75)
Prior radiotherapy, *n* (%)	13 (52)	23 (62)	6 (50)
Prior systemic therapy, *n* (%)	25 (100)	37 (100)	12 (100)
Median number of prior systemic therapies (range)	5 (1–9)	4 (1–13)	3.5 (1–12)
Prior immunotherapy, *n* (%)	11 (44)	10 (27)	6 (50)
Median AGP concentration, mg/dL	117^b^		53.9

Abbreviations: AGP, alpha‐1 acid glycoprotein; ECOG PS, Eastern Cooperative Oncology Group performance status.

^a^ At screening.

^b^ Median AGP concentration for Parts A and B of study NCT03166631 combined.

Thirty‐seven patients were treated in part B (50 mg QD, *n* = 6; 200 mg QD, *n* = 14; 400 mg QD, *n* = 8; 200 mg BID, *n* = 9). As with part A, patients were predominantly female and Caucasian (Table 1). All patients had received prior systemic therapy and were generally heavily pretreated; the median number of previous therapies was 4 (range 1–13); 10 patients (27%) had received prior immunotherapy.

In part A of the Japan study, 12 Asian patients received BI 891065 (100 mg QD, *n* = 3; 200 mg QD, *n* = 3; and 200 mg BID, *n* = 6). All patients had received prior systemic therapy; the median number of prior therapies was 3.5 (range 1–12). Six patients (50%) had received prior immunotherapy.

### 3.2 DLTs and MTD

In the USA study, no DLTs were reported with BI 891065 monotherapy during the MTD evaluation period, or subsequently. The MTD was not reached. There were three DLTs with BI 891065 monotherapy in the Japan study: two cases of grade 3 elevated bilirubin (100 mg QD and 200 mg BID) and one case of grade 3 maculopapular rash (200 mg BID). Both cases of grade 3 elevated bilirubin were manageable with dose interruption and reduction; doses were reduced to 50 mg QD and 200 mg QD, respectively. No DLTs were reported outside of the MTD evaluation period. The MTD was not reached.

The combination of BI 891065 with ezabenlimab was only assessed in part B of the USA study. There was one DLT during the MTD evaluation period (grade 2 pneumonitis; 400 mg QD). Two DLTs were reported outside the MTD evaluation period (grade 2 pneumonitis [200 mg QD] and grade 3 increased international normalized ratio [200 mg BID]). The MTD was not reached.

### 3.3 Safety

Overall, the safety profile of BI 891065 monotherapy was similar across the two studies (Table 2). Treatment‐emergent adverse events (TEAEs) occurred in 96% and 100% of patients, in the USA and Japan studies respectively. In the USA study, the most common TEAEs were fatigue (36%), nausea (28%), and diarrhea (24%). In the Japan study, the most common TEAEs were elevated bilirubin (33%), increased alanine aminotransferase (ALT), increased aspartate aminotransferase (AST), anemia, and nausea (all 25%). Across both studies, 13 patients (52%) and five patients (42%) experienced a grade ≥ 3 TEAE; these included grade 3 anemia, hypoxia, and elevated blood bilirubin (all two patients each), grade 3 herpes zoster, maculopapular rash, and hypercalcemia (one patient each); grade 4 laryngeal hemorrhage (one patient); grade 5 renal failure, respiratory arrest and septic shock (one patient each).

**TABLE 2:** Treatment‐emergent and treatment‐related adverse events reported in patients receiving BI 891065 monotherapy in the USA study (NCT03166631) and the Japan study (NCT04138823).

Study	TEAEs^a^	*N* (%)	TRAEs	*N* (%)
NCT03166631 (USA; *n* = 25)	Any	24 (96)	Any	13 (52)
	Fatigue	9 (36)	Fatigue	7 (28)
	Nausea	7 (28)	Nausea	4 (16)
	Diarrhea	6 (24)	Diarrhea	3 (12)
	Cough	6 (24)	Anemia	2 (8)
	Anemia	4 (16)	Blood bilirubin increased	2 (8)
	Constipation	4 (16)	AST increased	2 (8)
	Blood bilirubin increased	4 (16)	Decreased appetite	1 (4)
	Myalgia	3 (12)	Myalgia	1 (4)
	Headache	3 (12)	Headache	1 (4)
	Vomiting	3 (12)		
	AST increased	3 (12)		
NCT04138823 (Japan; *n* = 12)	Any	12 (100)	Any	9 (75)
	Blood bilirubin increased	4 (33)	AST increased	3 (25)
	Nausea	3 (25)	ALT increased	3 (25)
	AST increased	3 (25)	Blood bilirubin increased	2 (17)
	ALT increased	3 (25)	Anemia	2 (17)
	Anemia	3 (25)	Rash	2 (17)
	Constipation	2 (17)	Lymphocyte count decreased	2 (17)
	Herpes zoster	2 (17)	Nausea	1 (8)
	Rash	2 (17)	Vomiting	1 (8)
	Gamma‐glutamyl transferase increased	2 (17)	Gamma‐glutamyl transferase increased	1 (8)
	Lymphocyte count increased	2 (17)		
	Vomiting	2 (17)		

Abbreviations: AE, adverse event; ALT, alanine aminotransferase; AST, aspartate aminotransferase; TEAE, treatment‐emergent adverse events; TRAE, treatment‐related adverse events.

^a^ In at least three patients in the USA study and in at least two patients in the Japan study.

Treatment‐related adverse events (TRAEs) with BI 891065 monotherapy occurred in 13 patients (52%) and nine patients (75%) in the USA and Japan studies, respectively (Table 2). The most common (≥ 10%) TRAEs in the USA study were fatigue (28%), nausea (16%), and diarrhea (12%). The most common TRAEs in the Japan study were increased ALT and increased AST (both 25%). Grade 3 TRAEs occurred in two patients (8%) and five patients (42%) in the USA and Japan studies, respectively; these were fatigue, increased bilirubin, and maculopapular rash. There were no grade 4 or 5 TRAEs. Across both studies, one patient discontinued treatment due to a TRAE (grade 2 maculopapular rash).

While increased bilirubin was seen in several patients, it was asymptomatic and was not related to hemolysis or liver toxicity; in one patient an increase in both the size of liver lesions and liver function enzymes was seen. There appeared to be a dose‐dependent increase in total bilirubin, primarily triggered by indirect bilirubin, with most cases being present in the highest dose cohort of BI 891065 monotherapy (400 mg).

Of the 37 patients treated with BI 891065 plus ezabenlimab in part B of the USA study, 36 had at least one TEAE (Table 3). The most common (≥ 25%) TEAEs were fatigue, nausea, increased bilirubin, vomiting, decreased appetite, and diarrhea. Grade ≥ 3 TEAEs occurred in 16 patients (43%), including three patients who experienced grade 3 blood bilirubin increase and fatigue, two patients who experienced grade 3 acute kidney injury and anemia, and two patients (5%) who experienced grade 4 AEs (hyponatremia and sepsis [*n* = 1], and Stevens–Johnson syndrome [*n* = 1]).

**TABLE 3:** Treatment‐emergent and treatment‐related adverse events reported in patients receiving BI 891065 in combination with ezabenlimab in the USA study (NCT03166631) (*n* = 37).

TEAEs^a^	*N* (%)	TRAEs	*N* (%)
Any	36 (97)	Any	30 (81)
Fatigue	22 (60)	Fatigue	11 (30)
Nausea	16 (43)	Blood bilirubin increased	11 (30)
Blood bilirubin increased	15 (41)	Nausea	8 (22)
Vomiting	11 (30)	Rash maculopapular	7 (19)
Diarrhea	10 (27)	Diarrhea	6 (16)
Decreased appetite	10 (27)	Vomiting	5 (14)
Pyrexia	9 (24)	AST increased	4 (11)
Chills	8 (22)	Decreased appetite	4 (11)
AST increased	8 (22)	Stomatitis	4 (11)
Urinary tract infection	7 (19)	Pyrexia	3 (8)
Rash maculopapular	7 (19)	ALT increased	3 (8)
Candida infection	6 (16)	Chills	2 (5)
ALT increased	6 (16)		
Headache	6 (16)		
Dizziness	6 (16)		
Dyspnea	6 (16)		

Abbreviations: ALT, alanine aminotransferase; AST, aspartate aminotransferase; TEAE, treatment‐emergent adverse events; TRAE, treatment‐related adverse events.

^a^ In at least six patients.

Thirty patients (81%) treated with BI 891065 plus ezabenlimab experienced a TRAE. The most common (≥ 10%) TRAEs were fatigue and increased blood bilirubin (both 30%), nausea (22%), maculopapular rash (19%), diarrhea (16%), vomiting (14%), stomatitis, AST increased, and decreased appetite (all 11%). As in part A, increases in bilirubin were asymptomatic and accompanied by elevated liver enzymes only in cases of documented increase in liver lesions. Seven patients (19%) experienced a grade 3 TRAE, the most common being fatigue (5%), and one patient was reported with a grade 4 TRAE (Stevens–Johnson syndrome).

Four patients (11%) who received BI 891065 (varying doses and schedules) plus ezabenlimab 240 mg discontinued treatment due to a treatment‐emergent or treatment‐related AE: one patient due to grade 2 pneumonitis (BI 891065 200 mg QD; related to both study treatments), two patients due to grade 2 diarrhea (related to both study treatments) and grade 2 hypoxia (BI 891065 400 mg QD; related to BI 891065 only), and one patient due to grade 3 mental status changes (BI 891065 200 mg BID; unrelated to either study treatment).

There was no evidence of cytokine release syndrome with BI 891065 either as monotherapy or combined with ezabenlimab. In both part A and part B of the USA study, none of the cytokines measured posttreatment exhibited clinically significant increases versus baseline levels. In part A, minor, dose‐independent increases of monocyte chemoattractant protein‐1, interleukin (IL)‐6, and IL‐10 were observed. In part B, some ezabenlimab‐attributable increases in IL‐2, interferon‐γ, C‐X‐C Motif Chemokine Ligand (CXCL)‐9, and CXCL‐10 were noted.

### 3.4 Pharmacokinetics

**FIGURE 1:**
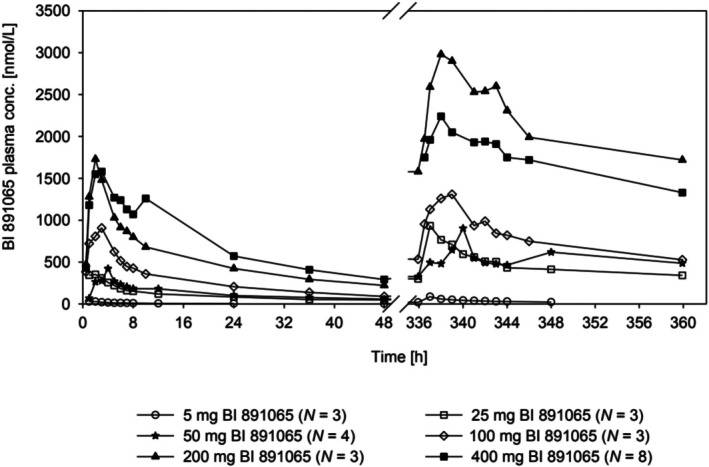
Plasma concentrations of BI 891065 over time after a single oral administration of BI 891065 (5–400 mg) monotherapy in part A of the USA study (NCT03166631). conc, concentration; h, hours.

After a single oral administration of BI 891065 (5–400 mg) monotherapy in part A of the USA study, C_max_ (36.1–2050.0 nmol/L) was reached after 1–3 h (Figure 1, Table 4). Terminal elimination half‐life (t_½_) was similar across dose groups (17.6–24.2 h). After multiple doses, C_max_ was 113.0–3070.0 nmol/L, and t_½_ was 27.8–51.5 h. C_max_, AUC_0–24_, and AUC_tau_ were dose proportional in the 5–400 mg dose range. Steady state was reached by Day 8 at the latest (168.0 h) by visual inspection. Interindividual variabilities of exposure (C_max_ and AUC) were moderate to high with an overlap of individual values between 25 and 50 mg, and between 200 and 400 mg; the geometric coefficient of variation ranged from 13.6% to 123.0%. Geometric mean exposures in the 200 mg dose group were higher than those seen in the 400 mg dose group.

**TABLE 4:** Pharmacokinetic parameters of BI 891065 monotherapy after a single oral administration in Part A of the USA study (NCT03166631I).

	*N*	Geometric means (gCV [%])						
		BI 891065 5 mg	BI 891065 15 mg	BI 891065 25 mg	BI 891065 50 mg	BI 891065 100 mg	BI 891065 200 mg	BI 891065 400 mg
Day 1								
C_max_, nmol/L	3/1/3/4/3/3/8	36.1 (78.9)	155	397 (80.0)	377 (67.3)	998 (55.9)	1730 (35.2)	2050 (57.7)
AUC_0‐24_, nmol·h/L	3/1/3/4/3/3/8	228 (65.9)	1400	3670 (101)	4010 (48.9)	9680 (66.3)	18,200 (13.6)	25,800 (50.8)
t_max_, median h (range)	3/1/3/4/3/3/8	1.1 (1.0–2.1)	2.1	1.2 (1.1–3.0)	3.0 (1.0–4.0)	3.0 (1.2–3.0)	2.1 (1.9–2.1)	2.9 (1.0–10.1)
t_1/2_, h	3/1/2/4/3/2/8	21.8 (25.0)	20.9	19.7 (30.7)	24.2 (28.7)	17.6 (15.1)	18.1 (22.7)	20.6 (14.4)
CL/F, mL/min	3/1/2/4/3/2/8	381 (53.2)	183	251 (35.1)	183 (24.2)	188 (80.3)	181 (186)	258 (52.8)
V_z_/F, L	3/1/2/4/3/2/8	718 (78.9)	330	428 (93.3)	384 (54.3)	287 (60.1)	283 (367)	460 (52.4)
Fe_0–24_, %	3/1/3/4/3/2/8	0.4 (17.5)	1.6	0.7 (30.1)	0.3 (56.5)	0.6 (123)	0.6 (0.3)	0.4 (75.5)
CL_R,0–24_, mL/min	3/1/3/4/3/2/7	2.5 (44.8)	4.8	1.3 (91.5)	1.2 (98.6)	1.9 (36.7)	1.8 (1.2)	2.1 (44.9)
Day 15								
C_max,ss_, nmol/L	2/1/3/4/3/3/6	113 (65.9)	163	948 (61.5)	708 (123)	1410 (56.0)	3070 (88.3)	2550 (68.5)
AUC_tau,ss_, nmol·h/L	2/1/2/4/3/3/5	900 (565)	2070	4420 (25,400)	10,700 (91.5)	18,800 (72.2)	51,300 (87.5)	48,500 (57.5)
t_max,ss_, median h (range)	2/1/3/4/3/3/6	1.2 (1.0)	3.1	1.4 (1.0–1.8)	2.0 (1.0–3.0)	2.3 (1.1–3.1)	2.0 (1.0–6.1)	2.0 (0.9–5.0)
R_A,Cmax_ (%)	2/1/3/4/3/3/6	1.4 (3.2)	1.1	2.4 (40.3)	1.9 (41.4)	1.4 (50.2)	1.8 (67.0)	1.4 (106)
R_A,AUCtau_ (%)	2/1/2/4/3/3/5	2.0 (3.1)	1.5	2.5 (2.8)	2.7 (42.2)	1.9 (42.1)	2.8 (81.6)	1.7 (65.1)

Abbreviations: AUC, area under the concentration‐time curve; AUC_0–24_, AUC from 0 to 24 h; AUC_tau,ss_, AUC of the dosing interval at steady state; BID, twice daily; CL/F, apparent clearance; CL_R,0–24_, renal clearance from 0 to 24 h; C_max_, maximum plasma concentration; C_max,ss_, C_max_ at steady state; Fe_0–24_, fraction of drug excreted unchanged from 0 to 24 h; gCV, geometric coefficient of variation; h, hours; L, liters; mg, milligrams; min, minutes; mL, milliliter; nmol, nanomole; R_A_, accumulation ratio; t_1/2_, half‐life; t_max_, time to peak drug concentration; V_z_/F, apparent volume of distribution during terminal phase.

After single and multiple QD administrations of BI 891065 100 and 200 mg, exposures in Asian patients were approximately 50%–60% lower than those in Caucasian patients. Likewise, exposures in Asian patients were lower than those in Caucasian patients after a single BID administration of BI 891065 200 mg. However, results were inconsistent after multiple BID administrations. Exposure was approximately 30% higher in Asian patients (Table S2).

In part B of the USA study, BI 891065 exposures (C_max_ and AUC) at the 50, 200, and 400 mg dose levels were similar to the corresponding values observed in part A, with large variability between patients (Table 4 and Table S3). There was no effect from ezabenlimab on exposure to BI 891065. The effect of a standard continental breakfast on the exposure of BI 891065 was investigated after multiple‐dose administration of 50, 200 and 400 mg BI 891065 in part B of 1379.1. No food effect on exposure to BI 891065 was confirmed statistically (data not shown).

### 3.5 Pharmacodynamics

**FIGURE 2:**
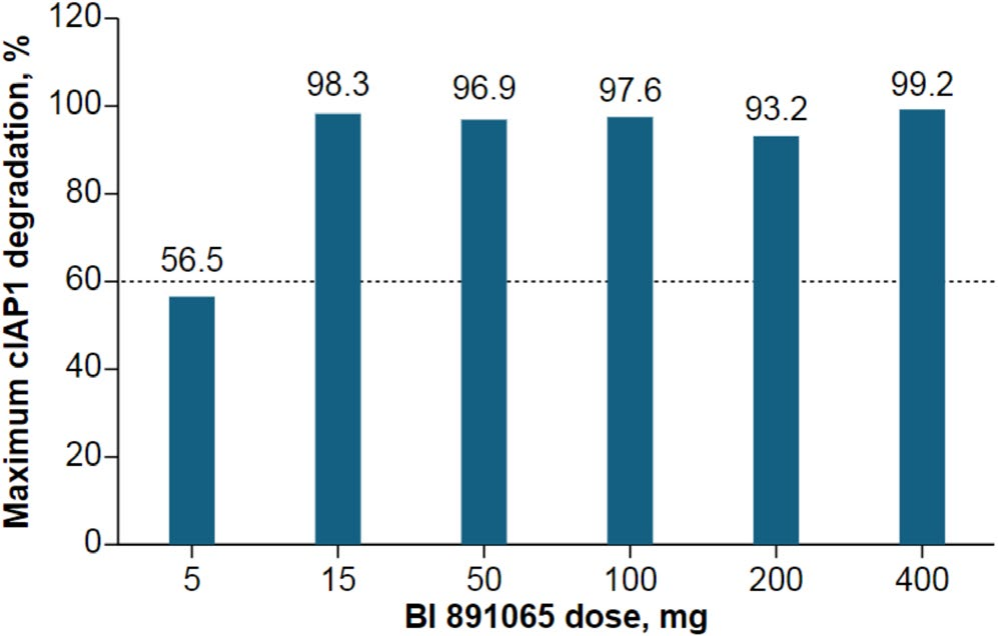
Maximum cIAP1 degradation* in PBMCs by BI 891065 dose in part A of the USA study (NCT03166631). cIAP1, cellular inhibitor of apoptosis 1; PBMCs, peripheral blood mononuclear cells. *The threshold to demonstrate cIAP1 degradation was 60%.

**FIGURE 3:**
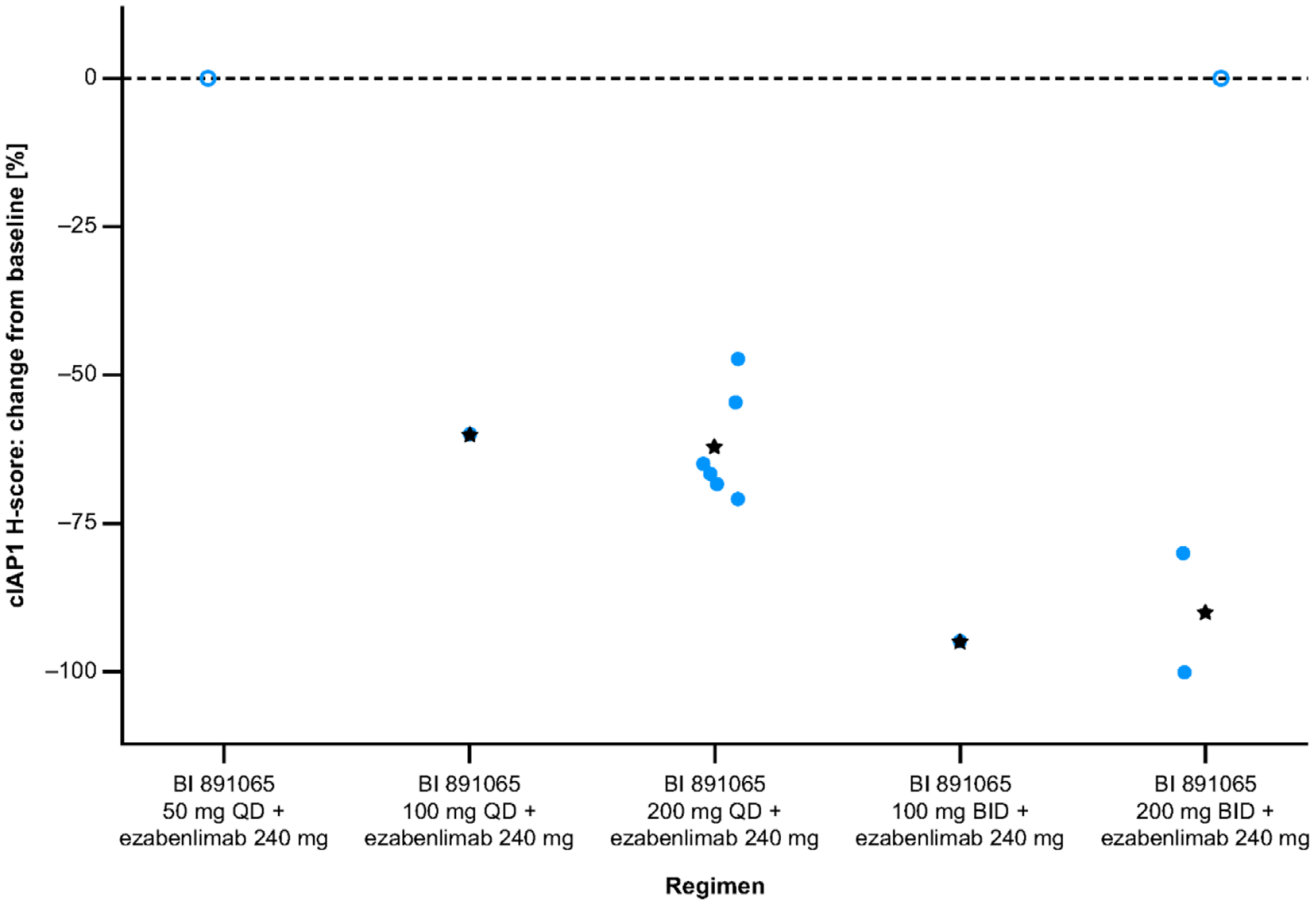
Percentage change from baseline of histochemical scoring assessment (H‐score) for cIAP1 tumor expression during treatment period (part B of the USA study). Two patients who received BI 891065 at a dose of 50 mg QD and 200 mg BID (one patient each) were off treatment for 14 and 3 days, respectively, before an on‐treatment biopsy was taken. Stars indicate mean for the group, circle indicates off‐treatment patients, who were excluded from the group in mean calculation. BID, twice daily; cIAP1, cellular inhibitor of apoptosis protein‐1; QD, once daily.

In part A of the USA study, 13 patients were evaluable for analysis of cIAP1 degradation in PBMCs (5 mg, *n* = 2; 15 mg, *n* = 1; 50 mg, *n* = 1; 100 mg, *n* = 2; 200 mg, *n* = 1; and 400 mg, *n* = 6). Of these patients, 11 (85%) demonstrated a > 60% reduction in cIAP1 levels (defined as the threshold by assay and analyte stability). The two patients who did not achieve the threshold reduction in cIAP1 had been treated at the 5 mg dose level (Figure 2). These results indicate that, for doses > 50 mg, BI 891065 is an active SMAC mimetic in the blood. In part B, paired pre‐ and post‐treatment tumor biopsy samples were available in 10 patients (50 mg QD, *n* = 1; 100 mg QD, *n* = 1; 200 mg QD, *n* = 6; 100 mg BID, *n* = 1; and 200 mg BID, *n* = 3 [actual doses of BI 891065 received prior to on‐treatment biopsy]). In all cases, there was a reduction in cIAP1 protein levels (Figure 3). An apparent difference was observed between the QD and BID dosing schemes. While the former led to a 50%–75% decrease in the mean histochemical scoring assessment (H‐score), the latter achieved reductions of > 75% (Figure 3).

### 3.6 Efficacy

No patient achieved an OR in either study. Ten patients (40%) in the USA study and three patients (25%) in the Japan study treated with BI 891065 monotherapy achieved stable disease (SD); 13 patients (35%) achieved SD on BI 891065 plus ezabenlimab. Across both studies, six patients had SD lasting ≥ 4 months. Among the patients treated with BI 891065 monotherapy these included three patients with head and neck cancer (*n* = 1, 4.0+ months [censored]), ovarian cancer (*n* = 1, 8.8+ months [censored]) and sarcoma (*n* = 1, 11.8+ months, ongoing at data cut‐off [March 29, 2021]). In patients treated with BI 891065 plus ezabenlimab, one patient each with melanoma, prostate cancer, ovarian cancer had SD duration of 11.6, 5.7 and 4.1, respectively. In addition, two patients (one lung cancer and one sarcoma) had ongoing SD but were censored at 3.1+ months. Data for best change in target lesion size, change in target lesion size over time, and durations of response per patient are shown in Figures S1–S3.

## 4 Discussion

In these two studies BI 891065 demonstrated a generally manageable safety profile, both as monotherapy and in combination with ezabenlimab. None of the treated patients had an objective response. PK analysis indicated dose proportionality of exposure to BI 891065; nevertheless, careful interpretation of the data is warranted given the small sample sizes in each dose group and a large variability observed. Ezabenlimab did not impact exposure to BI 891065. Pharmacodynamic investigations indicated that BI 891065 was acting as expected in terms of target engagement.

The overall safety profile of BI 891065 was broadly similar to those of other SMAC mimetics, but with some differences. There was no evidence of a clinically significant increase in cytokines with BI 891065 as has been observed with other SMAC mimetics, such as LCL161 and birinapant. For example, in a phase 1 dose‐finding trial in patients with advanced solid tumors (*n* = 53), LCL161 was associated with dose‐limiting cases of cytokine release syndrome at higher doses (i.e., above the MTD). In patients receiving doses below the MTD, LCL161 was associated with increased circulating cytokine levels, including TNF. However, no patients receiving LCL161 had an OR in the study, possibly due to the lack of tumor sensitivity to TNF [5]. The lack of cytokine induction with BI 891065 might reflect the fact that it spares XIAP, unlike other SMAC mimetics [13, 14]. Another notable difference between BI 891065 and other SMAC mimetics is the absence of Bell's palsy cases, a side effect that has been reported with birinapant [14]. Patients in the Japanese study appeared to have higher rates of TRAEs with BI 891065 monotherapy (75%) than those in the USA study (52%) with higher rates of anemia (17% vs. 8%) and decreased lymphocyte counts (17% vs. 0%). Moreover, all three DLTs occurred in the Japanese study. The increased occurrence of TRAEs in Japanese vs. Western patients in early‐phase oncology trials has been observed previously [20, 21] and has been suggested to be due to allelic variants of the genes for drug‐metabolizing enzymes across different racial/ethnic groups, ultimately affecting drug PK/PD [22].

The increase in bilirubin seen in several patients was not related to hemolysis or liver toxicity; a plausible explanation for the observed increases is the inhibition of uridine diphosphate glucuronosyltransferase 1‐1 (UGT 1A1) by BI 891065 and its metabolites. This is supported by the fact that the first patient in the Japan study who developed a grade 3 increase in bilirubin had UGT 1A1 *6 and *28 homozygous mutations.

There was moderate to high interindividual variability in BI 891065 exposure (C_max_ and AUC) in part A of the USA study, with an overlap in individual PK parameters in the BI 891065 200 and 400 mg dose groups. One of the potential explanations for the high variability seen in PK parameters is the low solubility of BI 891065 at high doses, which may reduce absorption into the systemic circulation following oral administration.

Geometric mean exposures (C_max_, C_max,ss_, AUC, and AUC_tau,ss_) were consistently lower in Asian patients than in Caucasian patients after 100 and 200 mg QD administration. In vitro data indicate that BI 891065 is metabolized by cytochrome P450 (CYP)3A4 and hydrolytic enzymes with a minor contribution of flavin‐containing monooxygenases. Hence, BI 891065 is not metabolized by CYP2C9, CYP2D6, or CYP2C19 and is not a substrate of BCRP, OATP1B1, or OATP1B3. Thus, CYP enzymes and transporters with known ethnic differences are not involved in the major metabolic pathways for BI 891065 [23, 24], and could not account for the ethnic difference observed in PK parameters in our study.

A possible explanation for ethnic differences in exposure to BI 891065 is that plasma AGP levels, the main binding protein to BI 891065 in human plasma, were lower in Asians than in Caucasians in these studies. Across both studies, exposure to BI 891065 tended to correlate with baseline AGP levels in patients who received BI 891065 QD. Furthermore, baseline AGP levels were around 50% lower in Asian patients than in Caucasian patients treated with BI 891065 QD, while there was no apparent difference in AGP levels between Caucasian and Asian patients treated with BI 891065 BID. This observation is consistent with previous studies [25, 26] and suggests that differences in AGP levels, given its role as a key binding partner for BI 891065 may explain the lower exposure in Asian patients. Of note, there were no clear ethnic differences in BI 891065 exposure, or AGP levels, with 200 mg BID dosing, supporting the observation that lower exposure in Asians was only seen with QD and BID dosing. Further clinical data collection and investigations are necessary to elucidate the fundamental mechanism of ethnic PK differences observed with BI 891065.

While no patient achieved an objective response, extended stable disease (defined as ≥ 4 months) was seen in six patients. It is noteworthy that the lack of responses seen with BI 891065 in these studies could be partly because the populations enrolled were heavily pretreated (median 3.5–5 prior lines of therapy), had advanced‐stage cancers and were not screened for the presence or absence of the target molecule, cIAP1. Furthermore, the studies were designed to assess safety, tolerability, and PK/pharmacodynamics, and patients may have received a sub‐optimal dose of BI 891065, particularly during the dose‐escalation phase.

Data from preclinical models suggest that the combination of BI 891065 and an anti‐PD‐1 antibody is synergistic [14]. However, the preliminary efficacy data reported here showed no improved efficacy with the combination and do not support the synergistic efficacy of BI 891065 in combination with ezabenlimab. This may be partly due to the short treatment duration, or because some patients had prior exposure to immunotherapy. Of note, while one patient in the Japan study was still receiving treatment at the time of writing, recruitment for the two studies has been discontinued. Both studies were discontinued as the observed pharmacodynamic and clinical efficacy didn't demonstrate sufficient promise to justify the continuation of clinical development. Due to the early termination of the study, fewer patients were enrolled than originally planned. This reduction in sample size may impact the interpretability of the data, particularly with respect to the evaluation of anti‐tumor efficacy. This is especially relevant for Part C (the expansion cohort) of the USA study, which was not initiated before the study was terminated. PK and PD assessments were designated as exploratory from the outset and evaluated as planned in parts A and B of the USA trial. Overall, the smaller patient population limits the generalizability of the study findings.

## 5 Conclusions

The safety profile of BI 891065 is manageable when administered as monotherapy or in combination with ezabenlimab. No signals of clinical efficacy were observed, but pharmacodynamic analyses indicated target engagement. Despite no objective responses being seen, the manageable safety profile and target engagement suggest that selective IAP inhibitors, such as BI 891065, may have greater success than investigational therapies in the same class with broader selectivity. Thus, these results may help guide the development of future SMAC mimetics.

## Author Contributions

**Manish R. Patel:** formal analysis; investigation; methodology; supervision; writing – original draft; writing – review and editing. **Erika P. Hamilton:** investigation; project administration; resources; writing – original draft; writing – review and editing. **Ben George:** investigation; writing – original draft; writing – review and editing. **Gunther Kretschmar:** conceptualization; data curation; formal analysis; writing – original draft; writing – review and editing. **Akiko Harada:** formal analysis; writing – original draft; writing – review and editing. **Ralph Graeser:** formal analysis; investigation; methodology; project administration; supervision; writing – original draft; writing – review and editing. **Anastasia Eleftheraki:** data curation; formal analysis; methodology; writing – original draft; writing – review and editing. **Yoshifumi Tachibana:** investigation; project administration; writing – original draft; writing – review and editing. **Noboru Yamamoto:** investigation; writing – original draft; writing – review and editing.

## Funding

This work was supported by Boehringer Ingelheim.

## Ethics Statement

The clinical protocols (ClinicalTrials.gov identifiers NCT03166631 and NCT04138823) were approved by the Institutional Review Boards of the participating centers, in accordance with the principles of the Declaration of Helsinki.

## Consent

All patients provided written informed consent before participation in any trial procedures.

## Conflicts of Interest

M.R.P. reports a consulting or advisory role for Daiichi Sankyo/UCB Japan, Kura Oncology, Accutar Biotech, Nurix, Mitsubishi Tanabe Pharma, and Shivanka Research; and their institution has received research funding from Agenus, Boehringer Ingelheim, Celgene, Cyteir Therapeutics, Daiichi Sankyo, Genentech/Roche, Janssen, Kymab, Loxo, Macrogenics, Merck, Moderna Therapeutics, Pfizer, Prelude Therapeutics, Seven and Eight Biopharmaceuticals, Syndax, Artios, Novartis, Nurix, TeneoBio, Zymeworks, Olema, Adagene, Astellas, Accutar Biotech, Compugen, Immunogen, Blueprint Pharmaceuticals, Cullinan Oncology, Immune‐Onc Therapeutics, Immunitas, Ribon Therapeutics, Step Pharma, Bristol‐Myers Squibb/Celgene, Kineta, Hotspot Therapeutics, Conjupro Biotherapeutics, Allorion Therapeutics, Vividion Therapeutics, Georgiamune, Kura Oncology, AstraZeneca, Zai Lab, Abbvie, Avenzo, D3 Bio, OnCusp Therapeutics, and Apollo, Medlink. E.P.H. reports their institution has received research funding from AbbVie, Acerta Pharma, Accutar Biotechnology, ADC Therapeutics, AKESOBIO Australia, Amgen, Aravive, ArQule, Artios, Arvinas, AstraZeneca, AtlasMedx, BeiGene, Black Diamond, Bliss BioPharmaceuticals, Boehringer Ingelheim, Bristol‐Myers Squibb, Cascadian Therapeutics, Clovis, Compugen, Context Therapeutics, Cullinan, Curis, CytomX, Daiichi Sankyo, Dana Farber Cancer Inst, Dantari, Deciphera, Duality Biologics, eFFECTOR Therapeutics, Eisai, Ellipses Pharma, Elucida Oncology, EMD Serono, Fochon Pharmaceuticals, FujiFilm, G1 Therapeutics, Gilead Sciences, H3 Biomedicine, Harpoon, Hutchinson MediPharma, Immunogen, Immunomedics, Incyte, Infinity Pharmaceuticals, Inspirna, InventisBio, Jacobio, Karyopharm, K‐Group Beta, Kind Pharmaceuticals, Leap Therapeutics, Lilly, Loxo Oncology, Lycera, Mabspace Biosciences, Macrogenics, MedImmune, Mersana, Merus, Millennium, Molecular Templates, Myriad Genetic Laboratories, Novartis, Nucana, Olema, OncoMed, Oncothyreon, ORIC Pharmaceuticals, Orinove, Orum Therapeutics, Pfizer, PharmaMar, Pieris Pharmaceuticals, Pionyr Immunotherapeutics, Plexxikon, Prelude Therapeutics, Profound Bio, Radius Health, Regeneron, Relay Therapeutics, Repertoire Immune Medicine, Rgenix, Roche/Genentech, SeaGen, Sermonix Pharmaceuticals, Shattuck Labs, Silverback Therapeutics, StemCentRx, Stemline Therapeutics, Sutro, Syndax, Syros, Taiho, TapImmune, Tesaro, Tolmar, Torque Therapeutics, Treadwell Therapeutics, Verastem, Zenith Epigenetics, and Zymeworks; and honoraria paid to their institution for consulting and advisory roles from Accutar Biotechnology, Arvinas, AstraZeneca, BeiGene, Circle Pharma, Daiichi Sankyo, Entos, Gilead Sciences, Incyclix Bio, IQVIA, Janssen, Jazz Pharmaceuticals, Jefferies LLC, Johnson and Johnson, Lilly, Medical Pharma Services, Mersana Therapeutics, Novartis, Pfizer, Pyxis Oncology, Roche/Genentech, Samsung Bioepis, Shorla Pharma, Stemline Therapeutics, Tempus Labs, Theratechnologies, and Zentalis Pharmaceuticals. B.G. reports honoraria from Ipsen, Bristol Myers Squibb, Foundation Medicine, Taiho Oncology, BTG (Boston Scientific), Roche/Genentech, Astellas, and AstraZeneca; and their institution has received grants or funds from Roche/Genentech, Hoffman La‐Roche, Taiho Oncology, Toray, Hutchison Medipharma, Mirati Therapeutics, CARsgen, Glyconex, Helix Biopharma, Pfizer, Tvardi Therapeutics, Faeth Therapeutics, BioNTech, Transcenta, Trishula Therapeutics, and Elicio Therapeutics. A.H. is an employee of Nippon Boehringer Ingelheim Co. Ltd. G.K. is an employee of Boehringer Ingelheim Pharma GmbH & Co. KG. R.G. has nothing to disclose. A.E. is an employee of Boehringer Ingelheim Pharma GmbH & Co. KG. Y.T. is an employee of Nippon Boehringer Ingelheim Co. Ltd. N.Y. reports a consulting or advisory role for Boehringer Ingelheim, Chugai Pharma, Cimic, Eisai, Healios, Merck, Mitsubishi Tanabe, Noile‐Immune Biotech, and Rakuten Medical; has received honoraria from Chugai Pharma, Daiichi Sankyo/UCB Japan, and Eisai; and their institution has received grants or funds from AbbVie, Amgen, Astellas, AstraZeneca, Bayer, Boehringer Ingelheim, Bristol Myers Squibb, Carna Biosciences, Chiome Bioscience, Chugai Pharma, Cimic, Daiichi‐Sankyo, Eisai, Eli Lilly, Genmab, GSK, InventisBio, Janssen Pharma, Kaken, Kyowa Kirin, Pfizer, Merck, MSD, Novartis, ONO, Otsuka, Rakuten Medical, Shionogi, Sumitomo Pharma, Taiho, Takeda, Toray, and Bicycle Therapeutics.

## Supporting information


**Table S1:** Inclusion and exclusion criteria.
**Table S2:** Comparison of selected pharmacokinetic parameters by race. Data are shown as geometric means.
**Table S3:** Pharmacokinetic parameters of BI 891065 after a single oral dose of BI 891065 50 mg, 200 mg and 400 mg QD, and combination of BI 891065 200 mg BID with ezabenlimab 240 mg. Data are shown as geometric means.
**Figure S1:** Best percent change in target lesion size from baseline in the USA study Part A (A), Part B (B) and the Japan study (C).
**Figure S2:** Best percent change in target lesion size over time in the USA study Part A (A), Part B (B) and the Japan study (C).
**Figure S3:** Swimmer plot for response per RECIST version 1.1 in the USA study Part A (A), Part B (B) and the Japan study (C).

## Data Availability

To ensure independent interpretation of clinical study results and enable authors to fulfill their role and obligations under the ICMJE criteria, Boehringer Ingelheim grants all external authors access to clinical study data pertinent to the development of the publication. In adherence to the Boehringer Ingelheim Policy on Transparency and Publication of Clinical Study Data, scientific and medical researchers can request access to clinical study data when it becomes available on Vivli—Center for Global Clinical Research Data, and earliest after publication of the primary manuscript in a peer‐reviewed journal, regulatory activities are complete, and other criteria are met. Please visit Medical and Clinical Trials | Clinical Research | MyStudyWindow for further information.
