# Supplementary material for: The Second Mitochondria‐Derived Activator of Caspases Mimetic BI 891065 in Patients With Advanced Solid Tumors: Results From Two Phase I Studies
Source: Cancer Med. 2025 Dec 17;14(24):e71451. doi: 10.1002/cam4.71451 (PMC12712391; doi:10.1002/cam4.71451)

# Supplementary Materials

**Dose-limiting toxicities definitions**

Hematologic toxicities:

- For patients with solid tumors:
  - Any grade 5 toxicity
  - Neutropenia ≥ grade 4 lasting for > 5 days
  - Febrile neutropenia of any duration (absolute neutrophil count < 1.0 × 10^9^ cells/L and fever ≥ 38.5°C)
  - Neutropenia grade 3 with documented infection
  - Grade 4 thrombocytopenia, or grade 3 thrombocytopenia with bleeding or a requirement for platelet transfusions
  - Grade 4 anemia unexplained by underlying disease.

Non-hematological toxicities:

- Aspartate aminotransferase (AST) or alanine aminotransferase (ALT) > 3x upper limit of normal (ULN) and concurrent total bilirubin > 2x ULN without initial findings of cholestasis (e.g. findings consistent with Hy's law or the US Food and Drug Administration definition of potential drug-induced liver injury)
- ≥ Grade 4 AST or ALT of any duration
- Any ≥ grade 3 non-hematologic toxicity with the following exceptions:
  - Grade 3 immune-related adverse event that resolved to ≤ grade 1 or to baseline with immunosuppressive therapy within 2 weeks
  - Grade 3 fatigue that persisted < 7 days
  - Grade 3 rash that resolved to ≤ grade 1 within 2 weeks
  - Grade 3 or 4 elevation in serum amylase and/or lipase that was not associated with clinical or radiographic evidence of pancreatitis
  - Grade 3 electrolyte abnormality that lasted < 72 h, was not clinically complicated, and resolved spontaneously or responded to conventional medical intervention
  - Grade 3 nausea or vomiting that lasted < 48 h, and resolved to ≤ grade 1 either spontaneously or with conventional medical intervention
  - Alopecia
  - Grade 3 endocrine disorders (thyroid, pituitary, and/or adrenal insufficiency) that were managed with or without systemic corticosteroid therapy and/or hormone replacement therapy, and the patient was asymptomatic
  - Grade 3 tumor flare syndrome.
- Any grade 2 pneumonitis of any duration.
- Any grade 2 related uveitis, eye pain, or blurred vision that did not respond to topical therapy and did not improve to grade 1 severity within 2 weeks or required systemic treatment.
- **Bioanalytical method**
- Liquid/liquid extraction with methyl tert-butyl ether was used to extract BI 891065 from human plasma. Before the extraction, isotope-labelled drug was added as an internal standard. The organic layer was collected, transferred to a new plate, and evaporated to dryness, and the residue reconstituted with a 60:40:0.1 water:acetonitrile:formic acid mixture. Samples were analyzed by liquid chromatography with tandem mass spectrometry using a Raptor FluoroPhenyl column (Restek) with a gradient ammonium acetate/acetonitrile/water/formic acid mobile phase.
- Blank plasma (BioChemed) was used as a control. Standard calibrators were prepared by spiking plasma with BI 891065 solutions to achieve nominal analyte concentrations of 1–1000 nmol/L. Standard calibrators were freshly spiked on the day of extraction for all experimental runs. For validation samples, plasma was spiked with BI 891065 solutions to achieve nominal analyte concentrations of 1, 3, 50, 500 and 750 nmol/L; quality control samples were prepared by spiking plasma with BI 891065 solutions to achieve nominal analyte concentrations of 3, 50, 500, 750 and 5000 nmol/L. Aliquots of each pool were stored at −20°C and −80°C and thawed for use at room temperature.
- Data acquisition was performed using Analyst version 1.5.2 software. Regression and calculation of results and statistics were performed using Watson® LIMS version 7.5 SP1.

**Bayesian Logistic Regression Model (BLRM) with overdose control**

- The estimated probability of a dose-limiting toxicity (DLT) at each dose level from the model was summarized using the following intervals: under dosing [0.00–0.16], targeted toxicity [0.16–0.33] and over toxicity [0.33–1.00].
- The BLRM recommended dose for the next cohort was the level with the highest posterior probability of the DLT rate falling in the target interval [0.16 –0.33] among the doses fulfilling escalation with overdose control criteria.
- The MTD was considered reached if one of the following criteria was fulfilled:
  - The posterior probability of the true DLT rate in the target interval [0.16 –0.33] of the MTD was above 0.5, or
  - At least 18 patients (1397.1 part A) or 12 patients (1379.1 part B) or 12 patient (1379.6 part A) were treated in the dose escalation phase of the trial, of which at least 6 at the MTD.
- Toxicity information on BI 891065 from the 1379.1 Phase I study was incorporated in the prior distribution for dose escalation in 1379.6.

**SUPPLEMENTARY TABLE S1** | Inclusion and exclusion criteria.

| **Inclusion criteria** | **Exclusion criteria** |
| --- | --- |
| 1. Provision of signed and dated, written ICF in accordance with ICH/GCP and local legislation prior to any trial-specific procedures, sampling, or analyses  2. Patients ≥ 18 years of age at the time of signature of the ICF (NCT03166631); being of legal age, according to local legislation, at screening. No upper limit (NCT04138823)  3. Male or female patients. Those of reproductive age had to be ready and able to use highly effective methods of birth control during trial participation and for at least 6 months after the last administration of trial medication. A list of contraception methods meeting these criteria was provided in the patient information  4. ECOG score: 0 to 1  5. Life expectancy of at least 12 weeks after the start of the treatment according to the Investigator’s judgement  6. Patients with a confirmed diagnosis of advanced, unresectable, and/or metastatic solid tumors, who had failed standard treatment, or for whom no therapy of proven efficacy exists, or who were not amenable to standard therapies. Measurable lesions according to RECIST version 1.1 had to be present | 1. Major surgery (“major” according to the Investigator’s assessment) performed within 12 weeks prior to randomization or planned within 12 months after screening (e.g. hip replacement; NCT03166631 and NCT04138823); moderate surgeries, (“moderate” according to the Investigator's assessment), performed within 4 weeks prior to the first administration (NCT04138823, only)  2. Presence of other active invasive cancers other than the one treated in this trial within 5 years prior to screening, except appropriately treated basal cell carcinoma of the skin, or in situ carcinoma of uterine cervix, or other local tumors considered cured by local treatment  3. Patients who had to or wished to continue the intake of restricted medications or any drug considered likely to interfere with the safe conduct of the trial  4. Previous administration of BI 891065 or BI 754091 (NCT03166631); previous administration of BI 891065 or other SMAC mimetic/IAP inhibitors (NCT04138823)  5. Enrolled in another investigational device or drug trial at the time of this study start, or less than 30 days since ending another investigational device or drug trial(s), or receiving other investigational treatments  6. Treatment with any other anticancer drug within 4 weeks or within 5 half-life periods (whichever came earlier) prior to first administration of BI 891065  7. Persistent toxicity from previous treatments that had not resolved to ≤ grade 1 (except for alopecia)  8. Active, known or suspected autoimmune disease (except vitiligo or resolved asthma/atopy)  9. Interstitial lung disease (NCT03166631); history (including current) of interstitial lung disease or pneumonitis within 5 years (NCT04138823)  10. Any of the following cardiac criteria:   - Mean resting corrected QT interval (QTcF) > 470 msec (NCT03166631) or > 480 msec (NCT04138823) - Any clinically important abnormalities (as assessed by the Investigator) in rhythm, conduction, or morphology of resting ECGs, e.g. complete left bundle branch block, third degree heart block - Any factors that increase the risk of QTc prolongation or risk of arrhythmic events such as heart failure, hypokalemia, congenital long QT syndrome, family history of long QT syndrome or unexplained sudden death under 40 years of age, or any concomitant medication known to prolong the QT interval - Patients with an EF < 55% (NCT03166631) or < 50% (NCT04138823) or the lower limit of normal of the institutional standard were excluded. Only in cases where the Investigator (or the treating physician or both) suspected cardiac disease with negative effect on the EF would the EF be measured during screening using an appropriate method according to local standards to confirm eligibility (e.g. echocardiogram [ECHO], multigated acquisition scan [MUGA]). An historic measurement of EF no older than 6 months prior to first administration of study drug would be accepted, provided that there was clinical evidence that the EF value had not worsened since this measurement in the opinion of the Investigator or of the treating physician or both.   11. Out of range laboratory values are defined as:   - ALT and AST > 3x the ULN if no demonstrable liver metastases or > 5x ULN in the presence of liver metastases - Total bilirubin > 1.5x ULN, except for patients with Gilbert's syndrome, who were excluded if total bilirubin was > 3.0x ULN or direct bilirubin was > 1.5x ULN.   12. Human immunodeficiency virus (HIV) infection, acute or chronic viral hepatitis  13. Known hypersensitivity to the trial drugs or their excipients  14. Serious concomitant disease or medical condition affecting compliance with trial requirements, or which were considered relevant for the evaluation of the efficacy or safety of the trial drug, such as cardiac, neurologic, psychiatric, infectious disease, or active ulcers (gastrointestinal tract, skin), or laboratory abnormality that could have  increased the risk associated with trial participation or trial drug administration, and in the judgment of the Investigator made the patient inappropriate for entry into the trial  15. Chronic alcohol or drug abuse or any condition that, in the Investigator’s opinion, made them an unreliable trial patient or unlikely to complete the trial  16. Females who were pregnant, nursing, or who planned to become pregnant while in the trial and for at least 6 months after the last administration of trial medication (NCT03166631 and NCT04138823); females who were nursing could be enrolled if they stopped nursing. In this case, the patient could not resume nursing even after discontinuation of trial treatment (NCT04138823, only)  17. Males who planned to father a child while in the trial and for at least 6 months after the last administration of trial medication (NCT03166631, only)  18. Known presence of symptomatic central nervous system metastases, unless asymptomatic and off corticosteroids and/or anticonvulsant therapy for at least 2 weeks prior to start of treatment (NCT03166631); untreated brain metastasis(es) that might have been considered active. Patients with previously treated brain metastases may participate provided they were stable, i.e. without evidence of disease progression by imaging for at least 4 weeks prior to the first dose of trial treatment, any neurologic symptoms had returned to baseline, and there was no evidence of new or enlarging brain metastases (NCT04138823)  19. Systemic treatment with any immunosuppressive medication within 1 week prior to treatment start (steroids of maximum 10 mg prednisolone equivalent per day were allowed; topical and inhaled steroids were not considered as immunosuppressive)  20. Known EGFR, known ALK, or known ROS1 genomic tumor aberrations, unless disease had progressed following available EGFR or ALK targeted therapy (including osimertinib for EGFR T790M-mutated non-small cell lung cancer)  21. Out of range lab values, defined as:   - Absolute neutrophil count < 1.5 x 10^9^/L (< 1500/mm^3^) - Platelet count < 100 x 10^9^/L - Hemoglobin < 90 g/L (< 9 g/dL) - Creatinine > 1.5x ULN (patients could enter if creatinine was > 1.5x ULN and eGFR was > 30 mL/min/1.73 m^2^; Chronic Kidney Disease Epidemiology Collaboration equation); confirmation of eGFR was only required when creatinine was > 1.5x ULN (NCT03166631); serum creatinine > 1.5x ULN, as measured by enzymatic assay, IDMS Jaffe assay, or non-IDMS Jaffe assay. If serum creatinine was > 1.5x ULN, patient was eligible if concurrent estimated glomerular filtration rate was ≥ 30 mL/min/1.73m^3^, as measured or calculated by Chronic Kidney Disease Epidemiology formula (NCT04138823).   22. Patients with known leptomeningeal disease (NCT04138823, only) |

Abbreviations: ALK, anaplastic lymphoma kinase; ALT, alanine transaminase; AST, aspartate aminotransferase; ECG, electrocardiogram; ECOG, Eastern Cooperative Oncology Group; EF, ejection fraction; EFGR, epidermal growth factor receptor; eGFR, estimated glomerular filtration rate; GCP, good clinical practice; ICF, informed consent form; ICH, International Council for Harmonization of Technical Requirements for Pharmaceuticals for Human Use; IDMS, isotope dilution mass spectrometry; RECIST v1.1, Response Evaluation Criteria in Solid Tumors version 1.1; ROS1, ROS proto-oncogene 1; SMAC, second mitochondria-derived activator of caspases; ULN, upper limit of normal.

**SUPPLEMENTARY TABLE S2** | Comparison of selected pharmacokinetic parameters by race. Data are shown as geometric means.

|  | **Geometric means (gCV [%])** | | | | | | | | | | | | | | | | | |
| --- | --- | --- | --- | --- | --- | --- | --- | --- | --- | --- | --- | --- | --- | --- | --- | --- | --- | --- |
|  | **BI 891065 100 mg QD, gCV (%)** | | | | | **BI 891065 200 mg QD, gCV (%)** | | | | | | | | **BI 891065 200 mg BID, gCV (%)** | | | | |
|  |  | | | | | **Part A** | | **Part B** | |  | | **Part A** | **Part B** |  | | | | |
|  | ***N*** | **Caucasian** | ***N*** | **Asian** | **Fold change** | ***N*** | **Caucasian** | ***N*** | **Caucasian** | ***N*** | **Asian** | **Fold change** | **Fold change** | ***N*** | **Caucasian** | ***N*** | **Asian** | **Fold change** |
| C_max_ (nmol/L) | 3 | 998  (55.9) | 3 | 445  (8.1) | 0.45 | 3 | 1730  (35.2) | 8 | 1800  (76.5) | 3 | 761  (48.3) | 0.44 | 0.42 | 9 | 1710  (81.7) | 6 | 1270 (45.6) | 0.74 |
| AUC_0–24_ (nmol∙h/L) | 3 | 9680  (66.3) | 3 | 5090  (1.3) | 0.53 | 3 | 18,200  (13.6) | 8 | 23,300  (86.0) | 3 | 9410  (52.1) | 0.52 | 0.40 | 9 | 20,900  (69.4) | 6 | 15,500 (34.6) | 0.74 |
| C_max,ss_ (nmol/L) | 3 | 1410  (56.0) | 2 | 886–1120 | - | 3 | 3070  (88.3) | 7 | 3160  (79.8) | 3 | 1370  (15.8) | 0.45 | 0.43 | 6 | 3460  (79.5) | 4 | 4630 (65.6) | 1.3 |
| AUC_tau,ss_ (nmol∙h/L) | 3 | 18,800  (72.2) | 2 | 10,800– 12,200 | - | 3 | 51,300  (87.5) | 7 | 54,600  (91.0) | 3 | 20,500 (15.1) | 0.40 | 0.38 | 6 | 34,400  (88.0) | 4 | 47,200 (64.8) | 1.4 |

Abbreviations: -, missing; AUC, area under the concentration–time curve; AUC_0-24_, AUC from 0 to 24 hours; AUC_tau,ss_, AUC over the dosing interval at steady state; BID, twice daily; C_max_, maximum plasma concentration; C_max,ss_, C_max_ at steady state; gCV; geometric coefficient of variation; QD, once daily.

AUC_tau,ss_ was AUC_0–12_,_ss_ for 200 mg BID.

gCV is not shown in cases where *N* ≤ 2.

**SUPPLEMENTARY TABLE S3** | Pharmacokinetic parameters of BI 891065 after a single oral dose of BI 891065 50 mg, 200 mg and 400 mg QD, and combination of BI 891065 200 mg BID with ezabenlimab 240 mg. Data are shown as geometric means.

|  | ***N*** | **BI 891065**  **50 mg QD,  gCV (%)** | **BI 891065**  **200 mg QD,  gCV (%)** | **BI 891065**  **400 mg QD,  gCV (%)** | **BI 891065**  **200 mg BID,  gCV (%)** |
| --- | --- | --- | --- | --- | --- |
| **Day 1**  C_max_ (nmol/L) | 6/14/8/9 | 427 (50.3) | 1690 (62.2) | 2680 (57.8) | 1710 (81.7) |
| C_max,norm_ (nmol/L/mg) | 6/14/8/9 | 8.54 (50.3) | 8.47 (62.2) | 6.69 (57.8) | 8.53 (81.7) |
| AUC_0–24_ (nmol∙h/L) | 6/13/7/9 | 3590 (45.5) | 18,200 (89.4) | 30,000 (52.9) | 20,900 (69.4) |
| AUC_0–24,norm_ (nmol∙h/L/mg) | 6/13/7/9 | 71.7 (45.5) | 91.2 (89.4) | 74.9 (52.9) | 105 (69.4) |
| t_max_ (h)^†^ | 6/14/8/9 | 1.96 (0.73–3.07) | 2.00 (0.83–5.15) | 3.02 (1.93–7.92) | 2.85 (1.75–3.52) |
| **Day 15**  C_max,ss_ (nmol/L) | 5/9/3/6 | 623 (77.0) | 3010 (67.8) | 3220 (33.4) | 3460 (79.5) |
| C_max,ss,norm_ (nmol/L/mg) | 5/9/3/6 | 12.5 (77.0) | 15.1 (67.8) | 8.04 (33.4) | 17.3 (79.5) |
| AUC_tau,ss_ (nmol∙h/L) | 5/9/3/6 | 7260 (91.0) | 50,600 (78.0) | 44,000 (49.1) | 34,400 (88.0)^‡^ |
| AUC_tau,ss,norm_ (nmol∙h/L/mg) | 5/9/3/6 | 145 (91.0) | 253 (78.0) | 110 (49.1) | 172 (88.0) |
| t_max,ss_ (h)^†^ | 5/9/3/6 | 2.05 (0.97–4.92) | 2.98 (1.03–5.08) | 3.00 (2.08–3.02) | 2.55 (2.00–6.98) |

Abbreviations: AUC, area under the concentration–time curve; AUC_0–24_, AUC from 0 to 24 hours; AUC_0–24,ss_, AUC_0–24_ at steady state; BID, twice daily; C_max_, maximum plasma concentration; C_max,ss_, C_max_ at steady state; gCV, geometric coefficient of variation; h, hour; norm, dose normalized; QD, once daily; ss, steady state; t_max_, time to peak drug concentration; t_max_,_ss,_ t_max_ at steady state.

^†^Median (min–max). ^‡^AUC_tau,ss_ was AUC_0–12,ss_ for BID.

**SUPPLEMENTARY FIGURE S1** | Best percent change in target lesion size from baseline in the USA study Part A (A), Part B (B) and the Japan study (C)

A


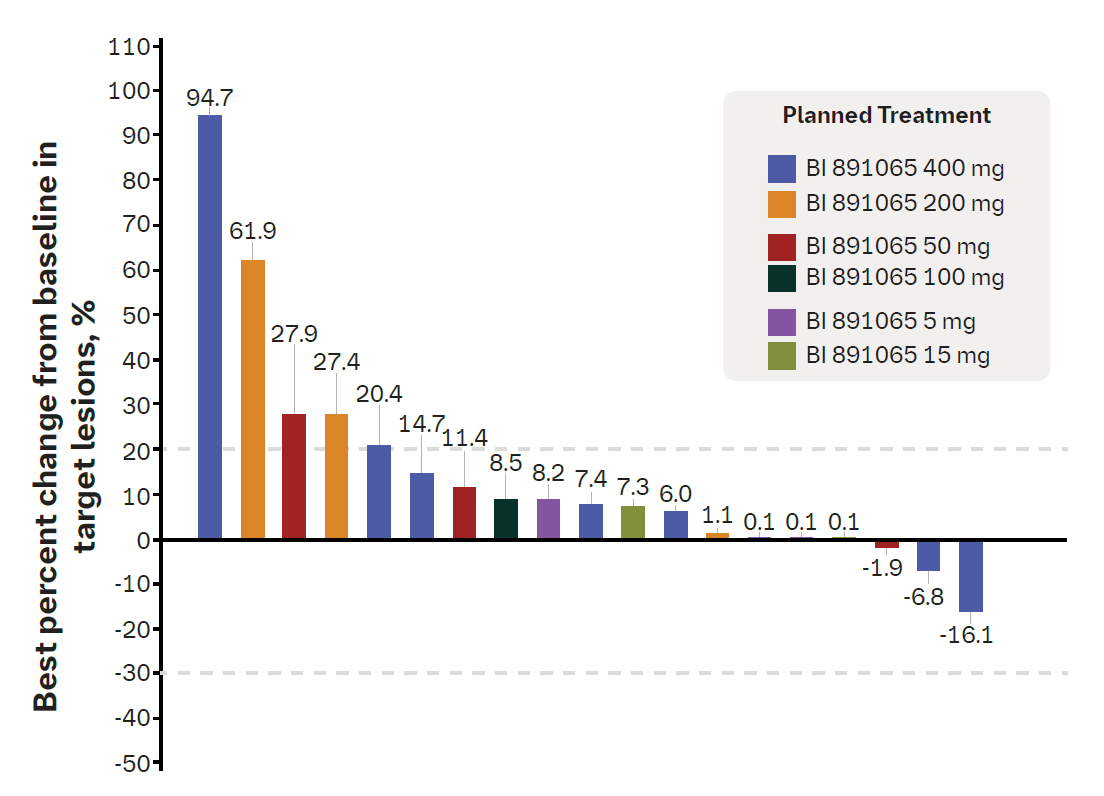


B


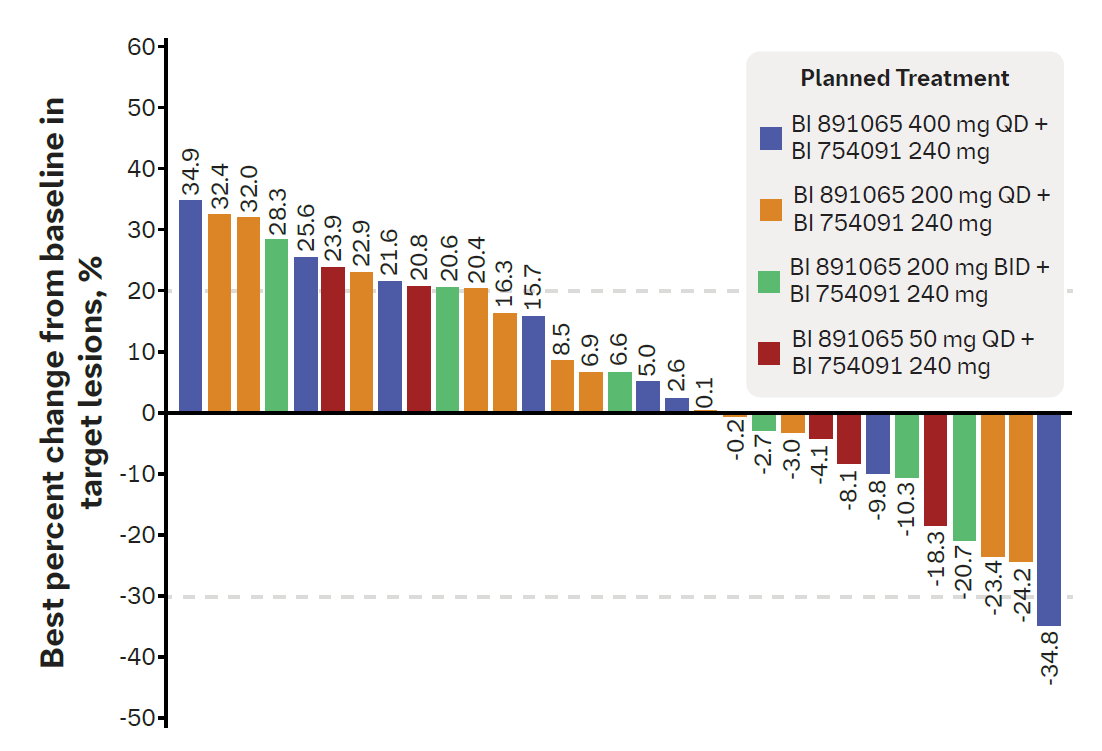


C


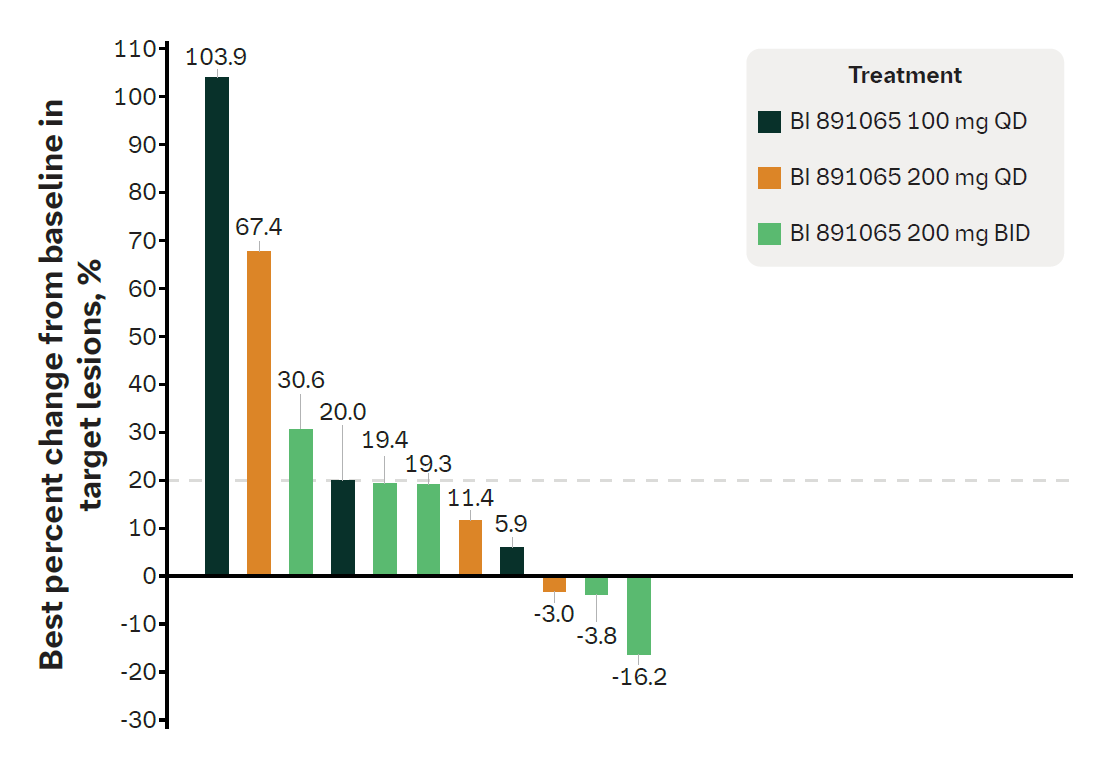


**SUPPLEMENTARY FIGURE S2** | Best percent change in target lesion size over time in the USA study Part A (A), Part B (B) and the Japan study (C)

A


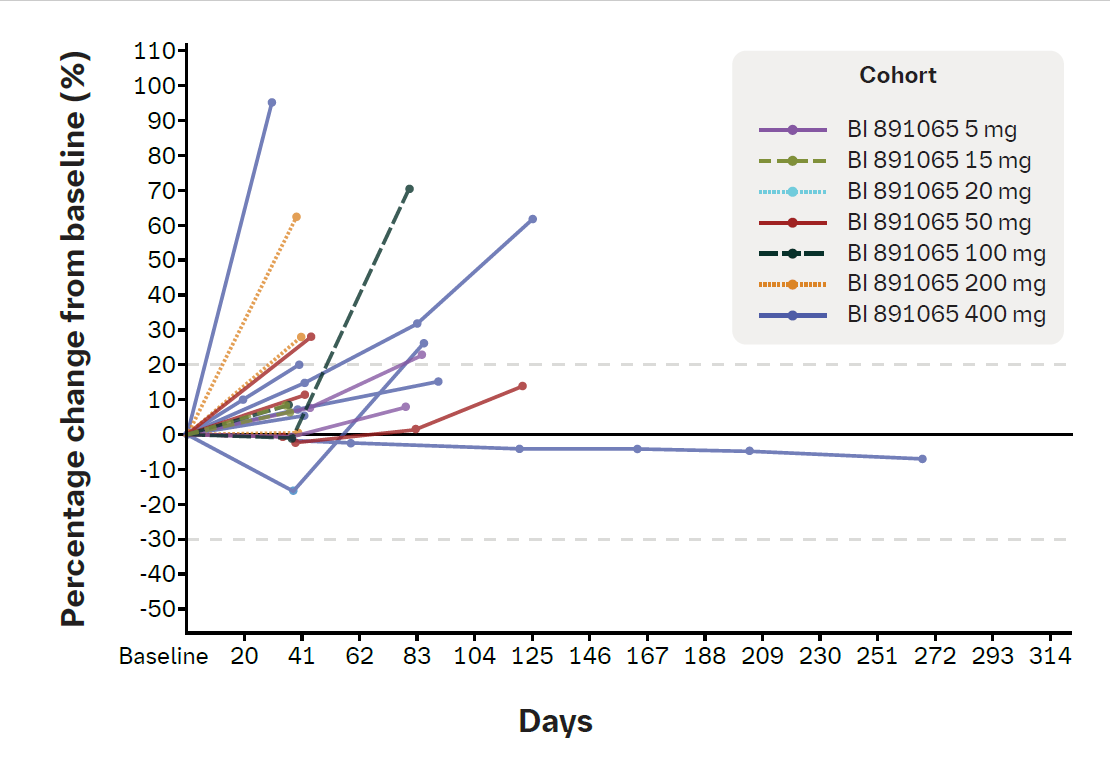


B


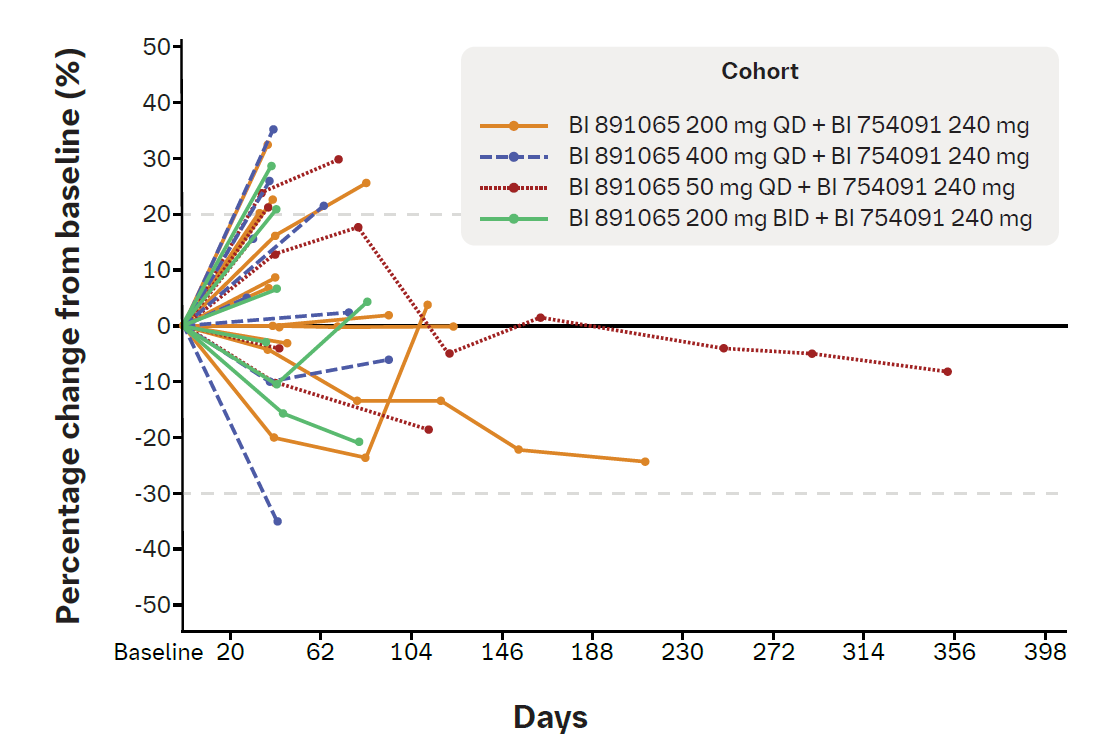


C


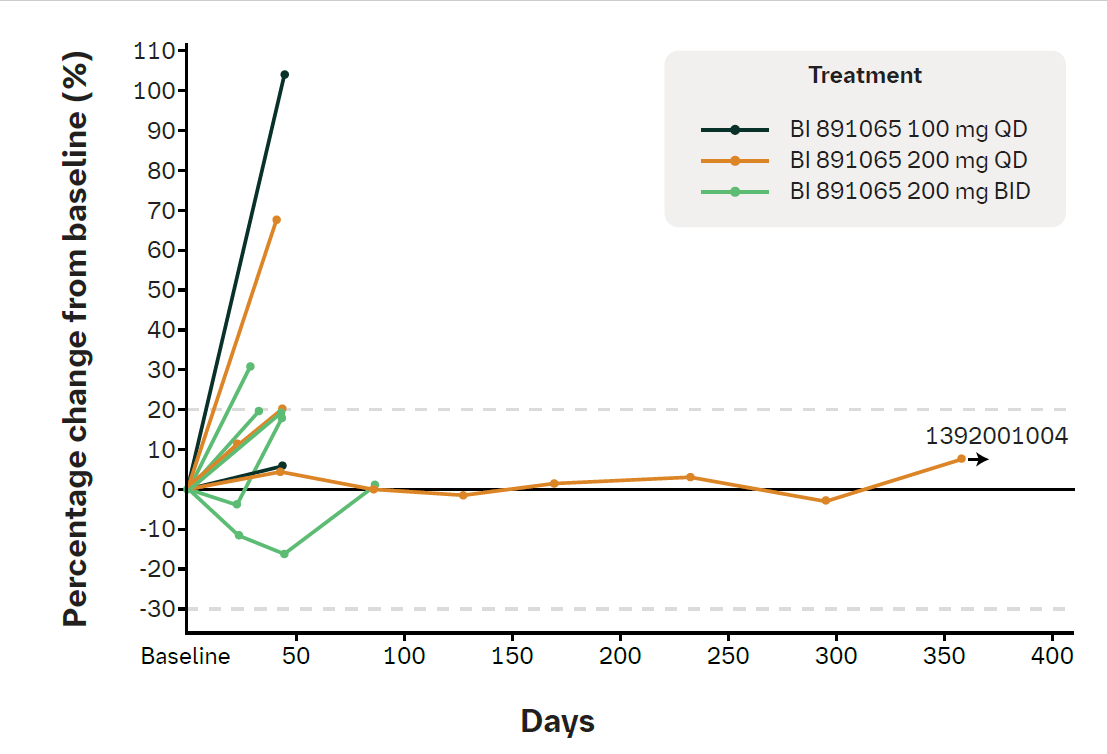


**SUPPLEMENTARY FIGURE S3** | Swimmer plot for response per RECIST version 1.1 in the USA study Part A (A), Part B (B) and the Japan study (C)

A


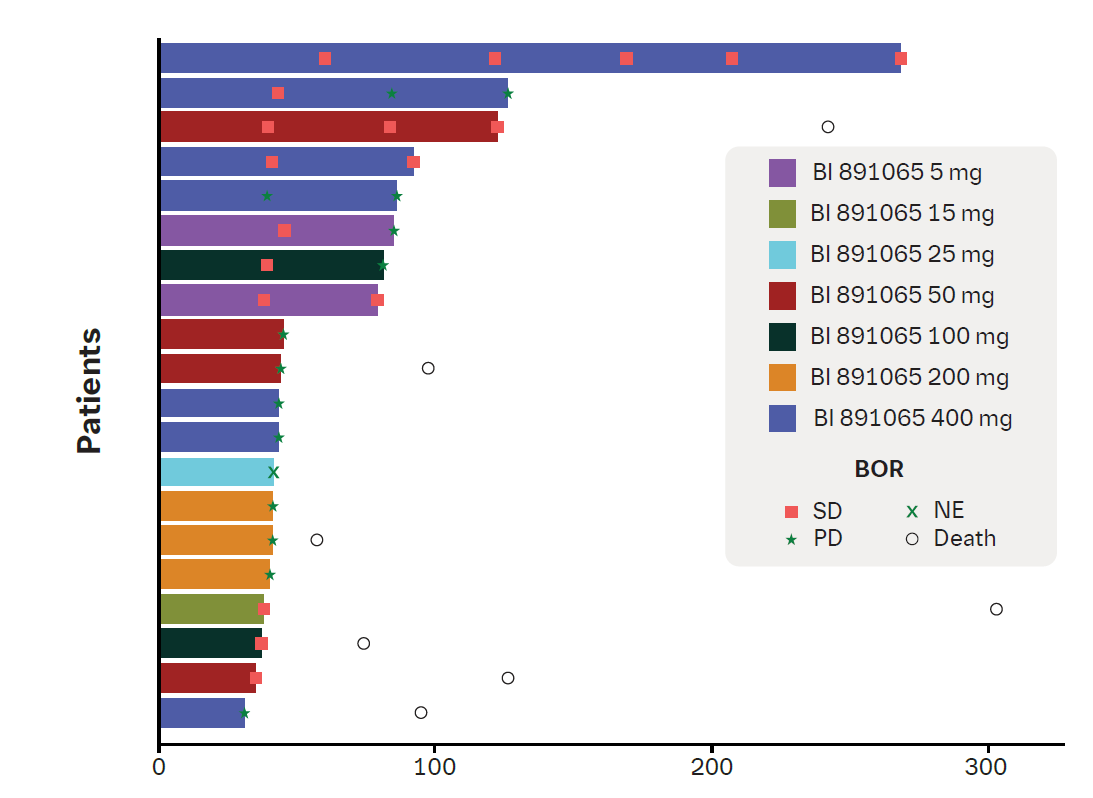


B


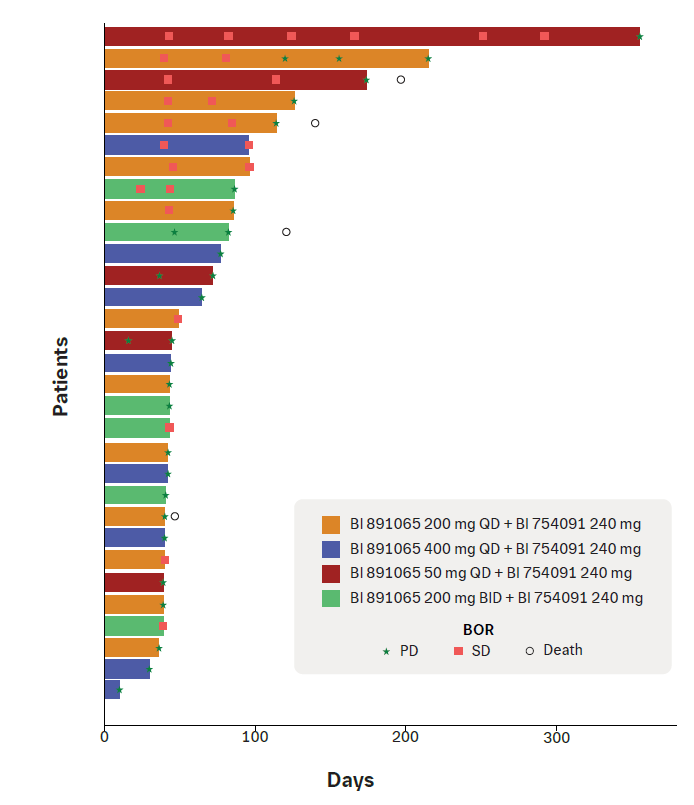


C


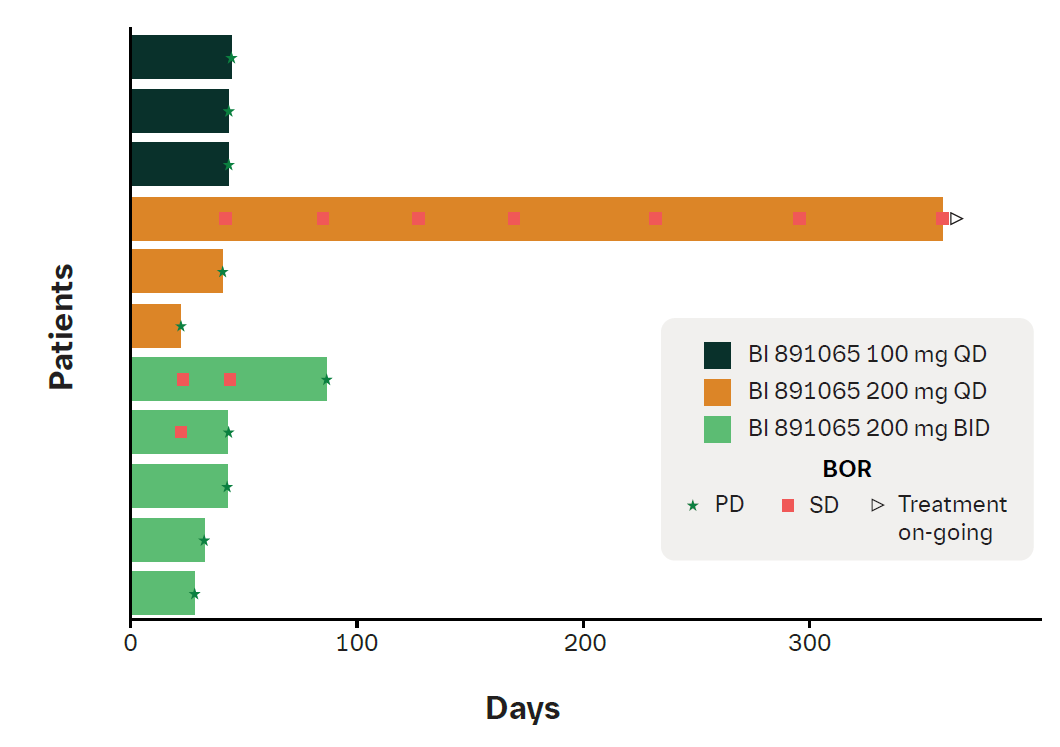

Supplement: Supplementary file 1 — Table S1: Inclusion and exclusion criteria. Table S2: Comparison of selected pharmacokinetic parameters by race. Data are shown as geometric means. Table S3: Pharmacokinetic parameters of BI 891065 after a single oral dose of BI 891065 50 mg, 200 mg and 400 mg QD, and combination of BI 891065 200 mg BID with ezabenlimab 240 mg. Data are shown as geometric means. Figure S1: Best percent change in target lesion size from baseline in the USA study Part A (A), Part B (B) and the Japan study (C). Figure S2: Best percent change in target lesion size over time in the USA study Part A (A), Part B (B) and the Japan study (C). Figure S3: Swimmer plot for response per RECIST version 1.1 in the USA study Part A (A), Part B (B) and the Japan study (C). [file CAM4-14-e71451-s001.docx]
